# Successful treatment with refractory myasthenia gravis that developed after allogeneic hematopoietic stem cell transplantation: two case reports

**DOI:** 10.3389/fimmu.2025.1539666

**Published:** 2025-02-27

**Authors:** Huili Zhang, Yi Wen, Yaotong Ou, Xi Chen, Yu Peng, Mingjun Lai, Wenjian Mo, Honghao Wang

**Affiliations:** ^1^ Department of Neurology, Guangzhou First People’s Hospital, School of Medicine, South China University of Technology, Guangzhou, China; ^2^ Department of Neurology, Guangzhou First People’s Hospital, School of Medicine, Guangdong Medical University, Zhanjiang, China; ^3^ Department of Hematology, Guangzhou First People’s Hospital, School of Medicine, South China University of Technology, Guangzhou, China

**Keywords:** myasthenia gravis, allogeneic stem cell transplantation, graft-versus-host disease, acetylcholine receptor antibody, muscle-specific kinase receptor antibodies

## Abstract

**Introduction:**

Myasthenia gravis (MG) is an autoimmune disorder caused by autoantibodies that target the neuromuscular junction, leading to muscle weakness and fatigability. Diagnosis is based on clinical presentation, confirmation of the presence of AChR-Ab, characteristic electromyography findings, and clinical improvement after administration of acetylcholinesterase inhibitors.MG is often associated with thymoma and other autoimmune diseases, but it is rare following allo-HSCT.

**Case report:**

we reports two rare cases of MG after transplantation, including the first case of post-transplantation double-antibody-positive MG. Patient 1 was a 45-year-old woman diagnosed with B-cell acute lymphoblastic leukemia. She underwent haploidentical allo-HSCT from a female donor (5/10 matching human leukocyte antigens [HLAs]) and developed graft-versus-host disease (GVHD) after transplantation. At 42 months after transplantation, the patient developed episodic generalized weakness, dysarthria, dysphagia, and axial weakness. The serum anti-acetylcholine receptor antibodies (AchR-Abs) level was > 20 nmol/L (normal range: < 0.4 nmol/L). She was diagnosed with MG type IIb according to the Myasthenia Gravis Foundation of America classification. At 44 months post-transplantation, the patient began to experience episodic cramps, Electromyography (EMG) revealed a small number of fibrillation potentials with the right thumb extensor and the right anterior tibial muscle in a relaxed state, as well as spastic discharge, considered indicative of cramp–fasciculation syndrome (CFS). Improvement was seen following treatment with carbamazepine. Patient 2 was a 49-year-old man diagnosed with acute myeloid leukemia. He underwent haploidentical allo-HSCT from his son and did not develop GVHD. At 23 months post-transplant, the patient experienced recurrent diplopia, ptosis, axial weakness, and dyspnea. Neostigmine and repetitive nerve stimulation tests were positive, the level of anti-AChR IgG antibodies and MuSK receptor antibodies were positive, leading to a diagnosis of type IVb MG. The symptoms were mostly relieved after rituximab treatment.

**Discussion:**

This article reports two rare cases of MG after transplantation, including the first case of post-transplantation double-antibody-positive MG, and reviews the general clinical characteristics of MG cases after allo-HSCT reported in previous literature. These cases enhance our understanding of MG following transplantation and add to the data on post-transplantation MG.

## Introduction

Myasthenia gravis (MG) is a acquired autoimmune disease mediated by acetylcholine receptor antibodies (AChR-Ab) at the neuromuscular junction postsynaptic membrane (1). It is characterized by generalized skeletal muscle fluctuating weakness and fatigue, with symptoms being more prominent in the morning and worsening after activity, but improving with rest or the use of anticholinesterase drugs. Diagnosis is based on clinical presentation, confirmation of the presence of AChR-Ab, characteristic electromyography findings, and clinical improvement after administration of acetylcholinesterase inhibitors. MG is often associated with thymoma and other autoimmune diseases, but it is rare following allo-HSCT. A previous study tracked 54 patients who underwent HSCT and found that 11 patients developed autoantibodies against acetylcholine receptors (2). Here, we report two cases of MG occurring after allo-HSCT for leukemia and review similar cases reported in the literature.

These cases enhance our understanding of MG following transplantation and add to the data on post-transplantation MG.

## Case description

**Figure 1:**
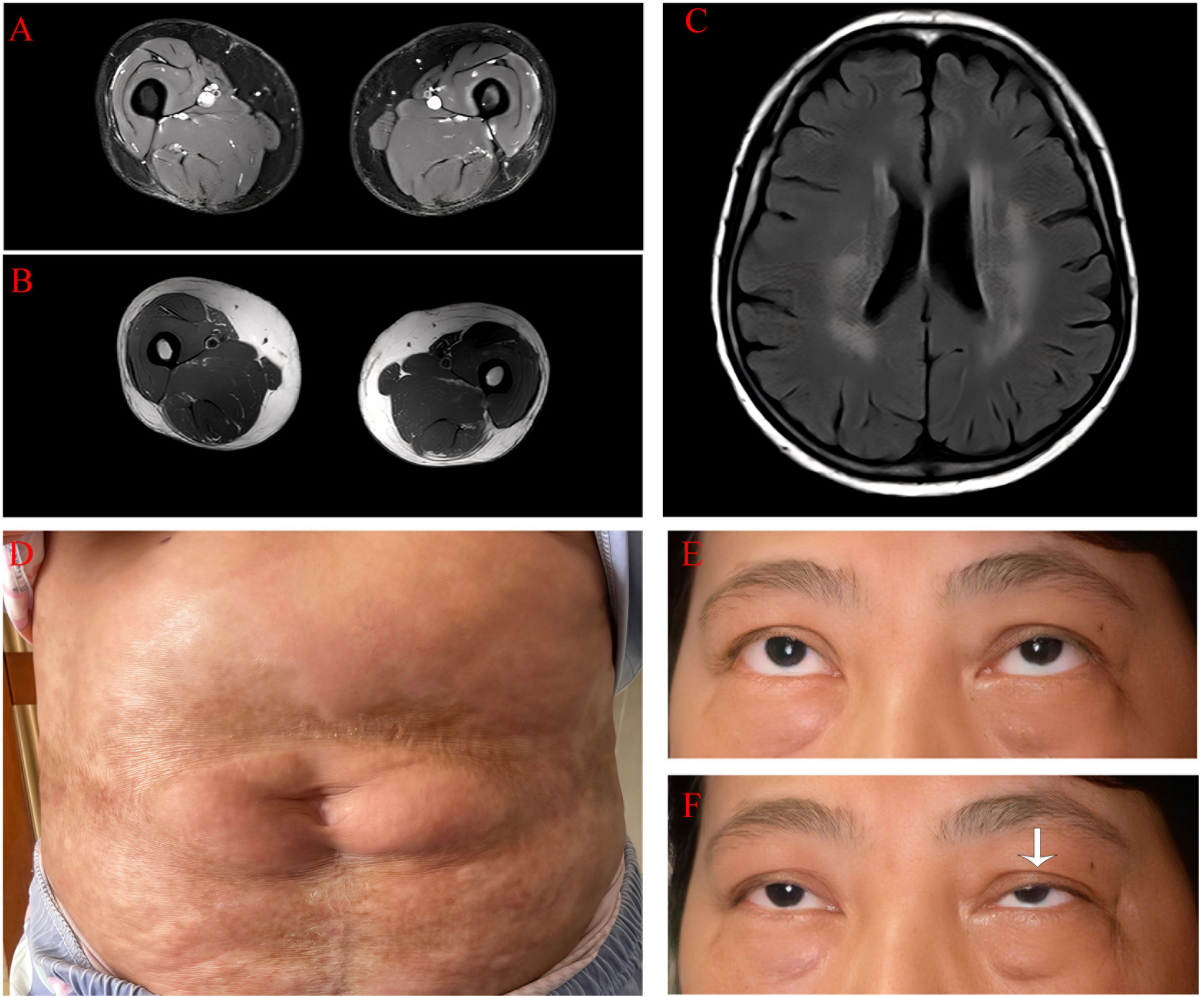
Clinical data of the patient 1. **(A)** There is no evidence of inflammatory edema in the axial T2 image of the thigh. **(B)** There is no fibrosis in the axial T1-weighted image of the thigh. **(C)** Bilateral, patchy, and abnormal signal intensities are observed on plain magnetic resonance imaging of the head, and the fluid-attenuated inversion recovery sequence shows high signal intensity. **(D)** In the lumbar and abdominal regions, there are observed band-like and patchy distributed areas of sclerotic atrophy of the skin with diffuse hyperpigmentation. The affected areas demonstrate surface depression and partial waxy luster. **(E)** The patient does not have ptosis. **(F)** After maintaining upward gaze for 5 minutes, the patient exhibited marked left-sided ptosis (as indicated by the white arrow).

Patient 1 was a 45-year-old woman diagnosed with B-cell acute lymphoblastic leukemia with TP53 gene mutation. After receiving vincristine, daunorubicin, cyclophosphamide, l-asparaginase, and prednisone (VDCLP) and hyperfractionated cyclophosphamide, vincristine, doxorubicin, and dexamethasone (Hyper-CVAD B) chemotherapy regimens, she underwent haploidentical allo-HSCT from a female donor (5/10 matching human leukocyte antigens [HLAs]) on December 12, 2018. One month after transplantation, the patient began experiencing acute graft-versus-host disease (GVHD) involving the skin, liver, and intestines. Methylprednisolone, cyclosporine A, and basiliximab were administered, regular follow-ups were conducted, and long-term immunosuppressive therapy was continued. At 4 months after transplantation, the patient presented with systemic symptoms including dry, wrinkled, and hardened skin, pigmentation deposition, and itching. Limb stiffness and inability to extend the knee and elbow joints were observed. The diagnosis was chronic graft-versus-host disease (cGVHD) involving the skin, muscle, and mucosa. Treatment with ibrutinib and ruxolitinib for graft rejection was initiated, but the response was poor, and symptoms continued to progress. With the patient’s informed consent, she was enrolled in a clinical trial on June 22, 2021, and began receiving the oral medication belumosudil. The patient’s skin itching and limb joint mobility improved. At 42 months after transplantation, the patient developed episodic generalized weakness, dysarthria, dysphagia, and axial weakness (see **Figure 1**). The serum anti-acetylcholine receptor antibodies (AchR-Abs) level was > 20 nmol/L (normal range: < 0.4 nmol/L). Chest computed tomography ruled out thymoma and thymic hyperplasia. Based on the patient’s comprehensive clinical information she was diagnosed with MG type IIb according to the Myasthenia Gravis Foundation of America classification. Treatment with prednisone (15 mg qd) and pyridostigmine significantly relieved symptoms after 2 months. At 44 months post-transplantation, the patient began to experience episodic cramps, initially confined to the fingers but gradually progressing to all four limbs. Eventually, the cramps occurred dozens of times per day. Electromyography (EMG) revealed a small number of fibrillation potentials with the right thumb extensor and the right anterior tibial muscle in a relaxed state, as well as spastic discharge, considered indicative of cramp–fasciculation syndrome (CFS). Improvement was seen following treatment with carbamazepine. Two years later, the patient’s myasthenic symptoms had completely resolved, and the frequency of cramp episodes decreased to three times per week. The patient continued to take oral methylprednisolone (20 mg qd) and carbamazepine, and the anti-AchR immunoglobulin G (IgG) antibodies level was maintained at 6.104 nmol/L according to ongoing follow-up tests.

**Figure 2:**
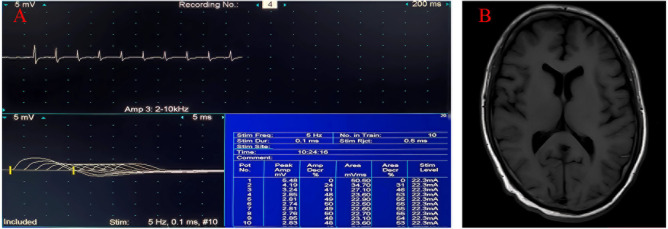
Clinical data of the patient 2. **(A)** Low-frequency stimulation of the left accessory nerve shows decreased waveform amplitude. **(B)** No abnormal signals are revealed by cranial magnetic resonance imaging.

Patient 2 was a 49-year-old man diagnosed with RUNX1::RUNX1T1-positive M2 acute myeloid leukemia. Chemotherapy included the IA (idarubicin/cytarabine) and MA (mitoxantrone/cytarabine) regimens, with subsequent CNS-directed intrathecal injection. He underwent haploidentical allo-HSCT from his son on February 1, 2020, with a donor–recipient HLA compatibility of 10/12. He did not develop GVHD. At 23 months post-transplant, the patient experienced recurrent diplopia, ptosis, axial weakness, and dyspnea. Neostigmine and repetitive nerve stimulation tests were positive, the level of anti-AChR IgG antibodies was 0.53 nmol/L (normal range: < 0.4 nmol/L), and MuSK receptor antibodies were positive at a titer of 1:100 (see **Figure 2**). Chest computed tomography ruled out thymoma and thymic hyperplasia, leading to a diagnosis of type IVb MG. Treatment with pyridostigmine, prednisone 60 mg qd, and intravenous immunoglobulin resulted in partial improvement, and the symptoms were mostly relieved after rituximab treatment. Two years later, the patient’s myasthenic symptoms had completely resolved, the anti-AChR IgG antibodies level was < 0.001 nmol/L, and MuSK receptor antibodies were negative. The patient continues to take azathioprine and is still in follow-up.

## Discussion

cGVHD is a late complication of allo-HSCT that may involve neuropathy presenting as myositis, immune-mediated neuropathy, cerebrovascular disease, central demyelinating disease, or immune-mediated encephalitis (3). MG is considered a rare neuromuscular manifestation of cGVHD.

The timing and course of MG after transplantation remain unclear. In most cases, MG symptoms occur gradually as immunosuppressive drugs are tapered off. It has been reported that most MG cases after bone marrow transplantation occur between 22 and 60 months post-transplantation, after most donor antibodies have been cleared from the recipient’s system (4). Additionally, in the two cases in this report, no MG symptoms were reported by the donors, and low-frequency repetitive nerve stimulation and serum AChR-Ab testing were negative, which is consistent with the low likelihood of MG occurring because of passive transfer of donor antibodies. A higher positivity rate of AChR-Abs was observed in patients with aplastic anemia and non-lymphocytic leukemia in 18 case reports (5). Specific risk factors for development of MG after allo-HSCT include mismatched donor–recipient sex and expression of specific HLA alleles, particularly HLA-Cw1, HLA-Cw7, and HLA-DR2 (6, 7). Although they were sex-matched and there was no expression of specific HLA alleles in the cases reported here, MG still developed after transplantation. However, the underlying mechanism remains unclear and requires further investigation.

CFS is a rare subtype of peripheral nervous system hyperexcitability syndrome with diverse etiologies involving mainly immune, tumor, or genetic factors. Clinical manifestations are non-specific and may include occasional muscle tremors, easily induced cramps, and muscle pain, without weakness symptoms (8, 9), as well as demonstrates fasciculation potentials and occasional myokymic discharges on EMG. In total, 19% of patients with CFS have voltage-gated potassium channel complex antibodies, primarily anti-contactin-associated protein 2 antibodies (Caspr2) and anti-leucine-rich glioma-inactivated 1 (LGI) antibodies (10). Membrane stabilizers (sodium channel blockers) can significantly alleviate symptoms (11). Patient 1 presented with episodic cramps without muscle hypertrophy or autonomic dysfunction. Caspr2 antibodies and LGI antibodies were negative, and EMG showed fibrillation potentials but no myokymic discharges. Symptoms significantly improved after oral carbamazepine administration. Based on comprehensive clinical information, the diagnosis was CSF. The instructions for belumosudil did not list cramp as a side effect. Moreover, the patient’s cramps did not change significantly even 2 weeks after discontinuation of belumosudil, suggesting that the condition might be transplant-related rather than belumosudil-induced. Besides, Head MRI shows extensive white matter lesions, but currently there is no evidence to suggest a correlation with cramps.

Patient 2 was positive for AchR antibodies and MuSK receptor antibodies; to our knowledge, this is the first example of double antibody positivity after transplantation in an MG patient. Double antibody positivity in MG without transplantation is extremely rare, with an occurrence rate of < 1% and a high rate of myasthenic crisis (12). Despite individual variability in the effectiveness of different types of immunosuppressive agents, double antibody-positive patients generally respond well to treatment with cholinesterase inhibitors and immunosuppressive agents, and the overall prognosis is good. After treatment with pyridostigmine bromide, prednisone and intravenous immunoglobulin were initiated in patient 2, the diplopia and ptosis improved, but dyspnea persisted until it was alleviated by treatment with rituximab. It is therefore worth considering that, if a patient does not respond well to conventional treatment for post-allo-HSCT MG, rituximab may be an additional treatment option. Strikingly, as the disease improved, further double antibody-positive tests were all negative.

Compared to idiopathic MG, the incidence of post-allo-HSCT MG is less than 1%. Patients often concurrently suffer from both aGVHD and cGVHD. The clinical features predominantly involve proximal limb muscle groups, typically affecting more than two muscle regions (13). Serological testing mainly shows positivity for AChR-Ab, while MuSK antibodies are rare. Therapeutically, there is a good response to pyridostigmine and immunosuppressive treatments, with some refractory cases requiring combined plasma exchange, intravenous immunoglobulin, or rituximab for intensified therapy. However, the majority of post-allo-HSCT MG patients experience symptom improvement and have a favorable overall prognosis. Due to the lack of long-term follow-up, the long-term prognosis cannot be assessed. Current case studies indicate no clear correlation between antibody titers and types with disease severity. Notably, none of the transplant-associated myasthenia gravis patients have been found to have thymoma or thymic hyperplasia, suggesting that the pathogenesis differs fundamentally from that of idiopathic MG. Further in-depth research is needed to elucidate the specific pathophysiological mechanisms.

Following HSCT, both patients experienced varying degrees of impact on their lives. Particularly for Patient 1, the sequential development of aGVHD and cGVHD, accompanied by scleroderma-like skin changes and muscle weakness, significantly affected her quality of life and psychological well-being. During GVHD treatment, the patient manifested depressed mood, loss of motivation, and even resistance to treatment, repeatedly expressing how myasthenic symptoms and restricted joint mobility severely affected her work and daily life. Through collaborative efforts between clinicians and family members, the patient eventually received treatment with bexarotene mesylate combined with psychological intervention, leading to symptom control and marked improvement in quality of life. During long-term follow-up, both patients consistently reported symptom fluctuations and personal perceptions of their conditions, profoundly reinforcing our understanding of the critical importance of preventing and managing post-transplant complications. These clinical observations underscore the essential significance of investigating the pathophysiological mechanisms underlying post-allo-HSCT MG.

Despite this study’s limitations, such as a small number of cases and a lack of in-depth exploration of the mechanisms underlying post-transplant MG, this study elucidated the clinical characteristics of two post-allo-HSCT MG cases. Common features included an association with immunosuppression, presence of other manifestations of cGVHD, a positive AChR-Ab test, absence of thymoma, and response to conventional treatments.

## Data Availability

The original contributions presented in the study are included in the article/**Supplementary Material**. Further inquiries can be directed to the corresponding authors.

## References

[1] Drachman DB . Myasthenia gravis. New Engl J Med. (1994) 330:1797–810. doi: 10.1056/NEJM199406233302507 8190158

[2] Smith CIE Hammarstr ML Lefvert AK . Bone-marrow grafting induces acetylcholine receptor antibody formation. Lancet. (1985) 325:978–. doi: 10.1016/S0140-6736(85)91742-8 2859427

[3] Oliver G Daniel W Hartmut B Hildegard G Jörn-Sven K Anita L . Neurological manifestations of chronic graft-versus-host disease after allogeneic haematopoietic stem cell transplantation: report from the consensus conference on clinical practice in chronic graft-versus-host disease. Brain A J Neurol. (2010) 10):2852. doi: 10.1093/brain/awq245 20846944

[4] Tse S Saunders EF Silverman E Vajsar J Becker L Meaney B . Myasthenia gravis and polymyositis as manifestations of chronic graft-versus-host-disease. Bone Marrow Transplant. (1999) 23:397. doi: 10.1038/sj.bmt.1701575 10100585

[5] Koeppen S Thirugnanasambanthan A Koldehoff M . Neuromuscular complications after hematopoietic stem cell transplantation. Support Care Cancer. (2014) 22:2337–41. doi: 10.1007/s00520-014-2225-0 24682581

[6] Mackey JR Desai S Larratt L Cwik V Nabholtz JM . Myasthenia gravis in association with allogeneic bone marrow transplantation: clinical observations, therapeutic implications and review of literature. Bone Marrow Transplant. (1997) 19:939–42. doi: 10.1038/sj.bmt.1700759 9156270

[7] Tsutsumi Y Kamiishi T Kikuchi R Ito S Matsuoka S Teshima T . Myasthenia gravis after allogeneic bone marrow transplantation: A case report and literature review. Hematol/Oncol Stem Cell Ther. (2017) 12:110–14. doi: 10.1016/j.hemonc.2017.04.001 28549768

[8] Poyraz M Matur Z Aysal F Tuzun E Hanoglu L Oge AE . Clinical, electrophysiological, and serological evaluation of patients with cramp-fasciculation syndrome. Noro Psikiyatri Arsivi. (2017) 54:183–6. doi: 10.5152/npa.2016.14816 PMC5491670 28680318

[9] Tahmoush AJ Alonso RJ Tahmoush GP Heiman-Patterson TD . Cramp,Fasciculation syndrome: A treatable hyperexcitable peripheral nerve disorder. Neurology. (1991) 41:1021–4. doi: 10.1212/WNL.41.7.1021 1648679

[10] Hoch W Mcconville J Helms S Newsom-Davis J Vincent A . Auto-antibodies to the receptor tyrosine kinase musk in patients with myasthenia gravis without acetylcholine receptor antibodies. Nat Med. (2001) 7:365. doi: 10.1038/85520 11231638

[11] Hurst RL Hobson-Webb LD . Therapeutic implications of peripheral nerve hyperexcitability in muscle cramping: A retrospective review. J Clin Neurophysiol Off Publ Am Electroencephalographic Soc. (2016) 33:560–3. doi: 10.1097/WNP.0000000000000291 27258601

[12] Poulas K Koutsouraki E Kordas G Kokla A Tzartos SJ . Anti-musk- and anti-achr-positive myasthenia gravis induced by D-penicillamine. J Neuroimmunol. (2012) 250:94–8. doi: 10.1016/j.jneuroim.2012.05.011 22683336

[13] Tsutsumi Y Kamiishi T Kikuchi R Ito S Matsuoka S Teshima T . Myasthenia gravis after allogeneic bone marrow transplantation: A case report and literature review. Hematol Oncol Stem Cell Ther. (2019) 12:110–4. doi: 10.1016/j.hemonc.2017.04.001 28549768

